# Isothiocyanates as effective agents against enterohemorrhagic *Escherichia coli*: insight to the mode of action

**DOI:** 10.1038/srep22263

**Published:** 2016-02-29

**Authors:** Dariusz Nowicki, Olga Rodzik, Anna Herman-Antosiewicz, Agnieszka Szalewska-Pałasz

**Affiliations:** 1 Department of Molecular Biology, University of Gdansk, Wita Stwosza 59, 80-308 Gdańsk, Poland

## Abstract

Production of Shiga toxins by enterohemorrhagic *Escherichia coli* (EHEC) which is responsible for the pathogenicity of these strains, is strictly correlated with induction of lambdoid bacteriophages present in the host’s genome, replication of phage DNA and expression of *stx* genes. Antibiotic treatment of EHEC infection may lead to induction of prophage into a lytic development, thus increasing the risk of severe complications. This, together with the spread of multi-drug resistance, increases the need for novel antimicrobial agents. We report here that isothiocyanates (ITC), plant secondary metabolites, such as sulforaphane (SFN), allyl isothiocyanate (AITC), benzyl isothiocynanate (BITC), phenyl isothiocyanate (PITC) and isopropyl isothiocyanate (IPRITC), inhibit bacterial growth and lytic development of *stx*-harboring prophages. The mechanism underlying the antimicrobial effect of ITCs involves the induction of global bacterial stress regulatory system, the stringent response. Its alarmone, guanosine penta/tetraphosphate ((p)ppGpp) affects major cellular processes, including nucleic acids synthesis, which leads to the efficient inhibition of both, prophage induction and toxin synthesis, abolishing in this way EHEC virulence for human and simian cells. Thus, ITCs could be considered as potential therapeutic agents in EHEC infections.

Among numerous toxins secreted by intestinal pathogens, Shiga toxins are ones of best described toxins that cause severe symptoms, including hemolytic colitis. First identified as produced by *Shigella dysenteriae*, they were reported to be produced by other bacterial species, mainly by Shiga toxin-producing *Escherichia coli* strains (STEC). Infections with *E. coli* STEC, including their specific sub-type identified as enterohemorrhagic *E. coli* (EHEC) strains comprise a significant danger to the public health as proven by recent foodborne and waterborne outbreaks and epidemics that also took place in developed countries, for example a STEC outbreak in Germany in 2011, or an EHEC outbreak in Japan the same year^1^,^2^,^3^,^4^,^5^. High level of pathogenicity of these bacteria is strictly correlated with production of enterotoxins. The presence of these virulence factors in human intestines results in a characteristic symptom of EHEC infections in humans, i.e. bloody diarrhea. Moreover, serious complications occur in 5–7% of patients, including hemolytic uremic syndrome (HUS). This severe disorder is characterized by hemolytic anemia, acute renal failure and thrombocytopenia, also leading to the damage to many internal organs^6^,^7^,^8^. Susceptibility to HUS increases for children and elderly patients. The mortality rate among HUS patients is as high as 10%, even with intensive treatment, indicating that HUS is a life-threatening disease, contributing to a high worldwide mortality of STEC infections. Thus, the threat of the infection by these pathogens, together with the increasing resistance to many of conventional antibiotics by pathogenic bacteria, justifies the necessity of the search for new efficient antimicrobial therapeutics. The most obvious target for the action of antibacterial compounds is the bacterial growth or survival. Various chemical compounds can stop growth of a bacterial culture, however, the therapeutic usage of such compounds depends on many factors, like potential toxicity for the human host, availability and transport in the human organism, or, for some specific types of pathogens, the undesirable effects of bacterial growth inhibition.

In STEC strains, the *stx* genes coding for Shiga toxins are located in the genomes of lambdoid bacteriophages, integrated into bacterial chromosome in the form of prophages. The virulence of STEC pathogenic strains depends on the production of Shiga toxins and is directly connected with the bacteriophage development. In these Shiga toxin- converting prophages, *stx* gene expression is under the control of pR’ promoter, which is active in the late phase of phage development. However, in the prophage state, the transcription from lytic-oriented phage promoters is repressed, including pR’ promoter, and subsequently Stx toxin production is also repressed. Thus, the efficient expression of *stx* genes and release of the toxin requires prophage induction. Bacteriophages can switch from the prophage status to the regular lytic development which leads to the excision of phage genome from bacterial chromosome, phage DNA replication and expression of phage genes, followed by phage-encoded protein synthesis, formation of phage particles and the lysis of bacterial cell. This is accompanied by the expression of toxin genes. Thus, all conditions that lead to the induction of lambdoid prophage harboring *stx* genes can result in the toxin production. Among these conditions are those that initiate SOS response, usually by damage to the bacterial DNA^9^. Many compounds and antibiotics can lead to DNA lesions as a result of DNA replication inhibition. It was demonstrated that prophage induction by exogenous (e.g. mitomycin C and antibiotics) and endogenous (e.g. hydrogen peroxide released from human neutrophils) stress factors, significantly increases the Stx production by their host strains^10^. Moreover, various compounds, including some antibiotics, can stimulate production of reactive oxygen species (ROS)^11^. Presence of ROS can result in DNA damage which in turn leads to the SOS response induction, and in case of prophages, excision of their DNA from a bacterial chromosome. Thus, any use of antimicrobial compounds to combat infections with Shiga toxin- producing *E. coli* strains has to be carefully considered and monitored.

Bacteria had evolved several global cellular responses to stress conditions. In addition to the SOS response, one of the most far-reaching is the stringent response. Its alarmones, unusual nucleotides, ppGpp and pppGpp (together referred to as (p)ppGpp), are synthesized under nutrient limitation (originally, (p)ppGpp was reported to be an effector of the response to amino acid starvation) and numerous physico-chemical stresses^12^. Stringent response regulates most of cellular processes, such as biofilm formation, quorum sensing, adaptation processes, bacterial virulence. *E. coli* has two main enzymes engaged in (p)ppGpp metabolism: synthetase I, encoded by the *relA* gene, responsible for ribosome-dependent production of (p)ppGpp upon amino acid starvation, and a bifunctional synthetase/hydrolase, product of *spoT* gene. The main aim of the stringent response is preventing unnecessary energy consumption during unfavorable conditions, that is inhibiting most of processes related to growth, e.g. stable RNA synthesis or DNA replication. (p)ppGpp exerts its main functions by affecting the transcriptional capacity of RNA polymerase, influencing transcription from numerous promoters. (p)ppGpp also affects development of bacteriophages, e.g. lambda phage and Shiga toxin-converting lambdoid phages, inhibiting phage DNA replication and lytic development^10^,^13^,^14^. Interestingly, as we previously demonstrated, one of the representatives of isothiocyanates (ITC), phenetyl ITC (PEITC), could inhibit growth of EHEC strains harboring *stx* genes and at the same time did not lead to the prophage induction and Shiga toxin production^15^. We also found that PEITC increased the level of (p)ppGpp in *E. coli* cells, both wild type and EHEC strains, to the extent observed in cells starved for amino acids. Thus, it was proposed that PEITC-mediated induction of the stringent response was responsible for inhibition of bacterial growth accompanied by inhibition of prophage induction and development, and Shiga toxin synthesis^15^. These findings put PEITC among the potential antimicrobial factors, especially in infections with pathogens when the standard antibiotic therapy is not advisable.

Isothiocyanates are compounds generated by hydrolysis of glucosinolates, especially abundant in cruciferous plants. ITCs, together with many other plant secondary metabolites, had attracted attention for many years due to their antibacterial, antimycotic, antiviral, anticancer, chemopreventive and antiparasitic properties, without conferring resistance to target microbes^16^,^17^,^18^. The antimicrobial action of several described ITCs was suggested to be related to impairment of bacterial membrane integrity, or, generally, to disruption of major metabolic processes, however the mechanism of this effect is not yet fully elucidated^19^,^20^,^21^. Most of ITCs, especially those of known chemopreventive effects, are plant-derived, however there are some synthetic ITCs, such as phenyl isothiocyanate (PITC), which are poorly investigated. ITCs are generally considered as safe and non-toxic to the environment because of their abundance in nature. Conventional antimicrobial factors commonly exert bactericidal or bacteriostatic effects which may promote the development of multi-drug resistant strains. Thus, the possibility to employ natural compounds to overcome increasing antibiotic resistance depends on the knowledge of their molecular mechanisms of action. The aim of this work was to compare activities of isothiocyanates differing in the structure of the alkyl/aryl group towards STEC strains, in particular their influence on bacterial growth, Shiga toxin-converting prophage induction, and expression of the *stx* genes, and to elucidate the mechanism of their activity.

## Methods

### Bacterial strains and growth conditions

*Escherichia coli* strains employed in this study are listed in the Table 1. Bacteria were grown in LB liquid medium at 37 °C with aeration achieved by shaking, or plated on solid medium (LB supplemented with 1.5% bacteriological agar), and incubated overnight at 37 °C. For assessing the amino acid effects on *E. coli* sensitivity to ITC, MOPS minimal medium^22^ supplemented with a relevant amino acid (20 mM) was used. When necessary, media were supplemented with relevant antibiotics (chloramphenicol, 20 μg/ml or 2.5 μg/ml; kanamycin, 50 μg/ml). Serine starvation was induced by addition of serine hydroxamate (SHX) (to final concentration of 0.5 mg/ml) to bacterial culture grown in LB medium.

The prophage induction was assessed essentially as described^15^. Briefly, *E. coli* lysogenic strain was cultured in LB and when A_600_ reached 0.1, the inducing agents: mitomycin C (at the final concentration of 1 μg/ml) or hydrogen peroxide (1 μM), were added. The cultures were supplemented with ITCs (under conditions indicated) at this time. Samples were withdrawn every 30 min, phages were extracted by adding chloroform, followed by vortexing and centrifugation. Phage titration and assessment of the phage titer was carried out by double agar overlay method, with the addition of 2.5 mg/ml chloramphenicol to the bottom agar for better visualization of plaques formed on the bacterial lawn^23^.

### Measurement of DNA and RNA synthesis

The assessment of nucleic acid synthesis was performed as described^15^. Briefly, bacteria were grown in LB to A_600_ of 0.1, and the radioactive precursor of DNA or RNA synthesis, [^3^H]thymidine or [^3^H]uridine, respectively, was added at 5 μCi. Samples (50 μl) were placed on Whatmann 3 MM filter paper and transferred to ice-cold 10% trichloroacetic acid (TCA) for 10 min, washed in 5% TCA and in 96% ethanol. After drying of filters, radioactivity was measured in a MicroBeta scintillation counter (PerkinElmer, Wallac). Results (expressed in cpm) from three independent experiments were normalized to bacterial culture density (A_600_) and are presented as mean values with SD.

### Antimicrobial activity and determination of bacterial growth inhibition

The minimum inhibitory concentrations (MIC) were assessed by the twofold broth microdilution method according to BSAC standard methodology^24^ with the inoculum of 10^6^ cfu. The concentration range of the ITCs used was from 0.25 to 32 mM. The dilution representing MIC and concentrations above MIC were then plated on LB agar plates and viable cfu/mL were enumerated to obtain the minimum bactericidal concentration (MBC) after 20 hours of incubation in 37 °C. Zone inhibition measurement was performed according to Kirby-Bauer Disk Diffusion Susceptibility Test^25^. Sterile filter paper discs (6 mm in diameter) impregnated with 15 μl of the test chemicals or DMSO were placed on the agar plate seeded with the tested *E. coli* strain. Growth inhibition kinetics was determined spectrophotometrically (A_600_) in the presence of relevant concentrations of ITCs.

### Assessment of (p)ppGpp cellular levels

ppGpp and pppGpp levels were measured basically as described^22^ with modifications as in^15^. Briefly, overnight bacterial culture in MOPS minimal medium was diluted in the same medium, but with a low phosphate concentration (0.4 mM), grown to A_600_ of 0.2. Then, bacteria were diluted (1:10) in the same medium with the addition of [^32^P]orthophosphoric acid (150 μCi/ml), and grown for at least two generations. Next, at the time zero, ITCs or SHX were added. Bacterial samples, collected at specified time points, were lysed with formic acid (13 M) in three cycles of freeze-thaw procedure. After centrifugation, nucleotide extracts were separated by thin-layer chromatography, using PEI cellulose plates in 1.5 M potassium phosphate buffer (pH 3.4). The chromatograms were analyzed with a phosphorimager (Typhoon, GE Healthcare).

### Microscopic analysis

Fluorescent microscopy was performed as describe previously^15^, using Leica DMI4000B microscope fitted with a DFC365FX camera (Leica). Bacteria were cultivated the same way as in the phage induction experiment. SynaptoRed (Sigma Aldrich) was used at 10 μM, to stain cell membranes. Propidium iodide was used at 10 μM, to stain nucleic acids in the cell with disrupted cell membranes.

### Study of toxicity of bacterial lysates on HeLa and Vero cells

Measurement of bacterial lysate toxicity to human and simian cell lines was carried out as described previously^26^,^15^. Briefly, cell lines were cultivated in DMEM medium (Dulbecco, Gibco), pH 7.4, supplemented with 10% heat-inactivated fetal bovine serum (FBS), antibiotic and antimycotic solution (all purchased from Sigma, Germany), and incubated at 37 °C in a humidified 5% CO_2_ atmosphere. The MTT [3-(4,5-dimethylthiazol-2-yl)-2,5-diphenyltetrazolium bromide] colorimetric assay was performed to assess viable cells in a Vero and HeLa cell cultures after 48 h of treatment with the supernatant obtained from EHEC cultures, treated with mitC or ITCs at various concentrations, as indicated. Assay was performed in triplicate and repeated in three independent experiments.

### Measurement of the reactive oxygen species (ROS)

The production of ROS was assessed essentially as described before^27^,^28^. Briefly, bacteria were cultured in MOPS medium to A_600_ of 0.5. After washing cells three times with a PBS, fluorescent probe 2,7-dichlorofluorescein diacetate (DCFH-DA) was added to a final concentration of 30 μM. After 60 min incubation with shaking at 37 °C in the dark, bacteria were centrifuged, and washed twice with PBS Bacteria were concentrated ~10-fold in PBS and treated with kanamycin (at 10 μg.ml), ampicillin (2.5 μg/ml), hydrogen peroxide (1 μM) or 1× MIC of ITCs (as indicated) for 2 hours at 37 °C. The fluorescent signal was measured with a fluorescence spectrophotometer at the excitation wavelength of 488 nm and emission wavelength of 535 nm. The results were normalized to cell growth (A_600_).

### Statistical analyses

Significant differences among the results were examined by the T-student test. The data were expressed as the means ± SD. In experiments devoted to determine toxicity of bacterial lysates to human or simian cells, one-way analysis of variance (ANOVA) with Bonferroni’s post hoc test was also employed. Differences were considered significant when P values were <0.05.

## Results

### ITCs inhibit both, the bacterial growth and stx-harboring prophage lytic development

**Figure 1:**
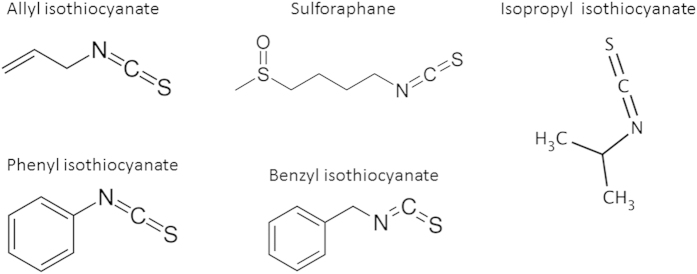
The chemical structures of ITCs employed in this study.

**Figure 2:**
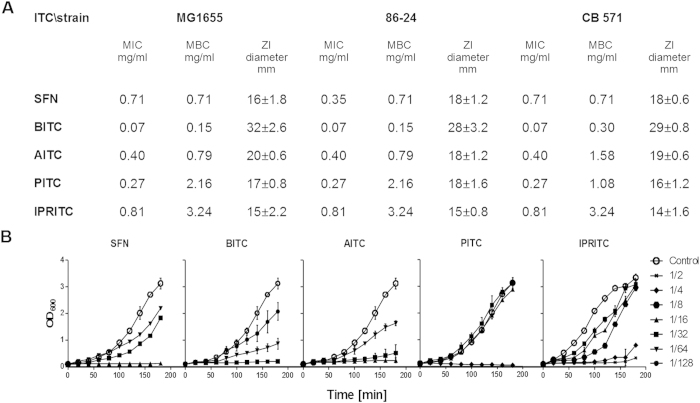
Effect of ITCs on bacterial growth. (**A**) MIC, MBC and zone inhibition were determined for each ITC. The results are from at least three independent experiments (**B**) *E. coli* strain MG1655 was cultivated in the absence or in the presence of ITCs (SFN, BITC, AITC, PITC, IPRITC). Concentrations of ITC are presented in relation to MIC determined as in A.

The chemopreventive effects of the isothiocyanates could involve direct interaction with cellular proteins, as was reported for sulforaphane (SFN) and PEITC^28^. It is plausible, that the different spatial distribution of residues and isothiocyanate moiety could play an important role in the interaction with the cellular targets. Thus, we compared the antibacterial properties of various isothiocyanates. For our studies, we employed the following ITCs: SFN, allyl isothiocyanate (AITC), benzyl isothiocynanate (BITC), phenyl isothiocyanate (PITC) and isopropyl isothiocyanate (IPRITC) (Fig. 1). All of them, except for IPRITC, are plant-derived natural compounds. The ITCs were selected based on various chemical structures of their side groups, where only the –N = C = S isothiocyanate chemical group remained common. To evaluate their effects on bacterial growth the minimum inhibitory concentration (MIC) and the minimum bactericidal concentration (MBC), as well as the zone inhibition diameter were assessed for the clinical isolates O157:H7strains: CB571, 86–24 and wild type *E. coli* MG1655 strain. Then, the subinhibitory concentrations (at twofold dilution intervals) were used in the assessment of ITC’s effect on log phase of *E. coli* growth in the liquid culture. We observed that the growth inhibition varied for different ITCs, with the most potent effect of BITC which was evident already at 1/128 MIC while the growth arrest during first hours of cultivation was observed at 1/32 MIC or other tested ITCs, higher concentrations were necessary to obtain full growth arrest at the exponential growth phase, e.g. 1/16 for SFN and AITC, 1/4 for PITC or 1/2 for IPRITC (Fig. 2B). Importantly, the growth inhibition by ITCs was very similar for wild type *E. coli* as well as for strains carrying Shiga toxin-converting prophages (Fig. 2A). The only minor exception was MIC observed for the effect of SFN on 86–24 strain.

**Figure 3:**
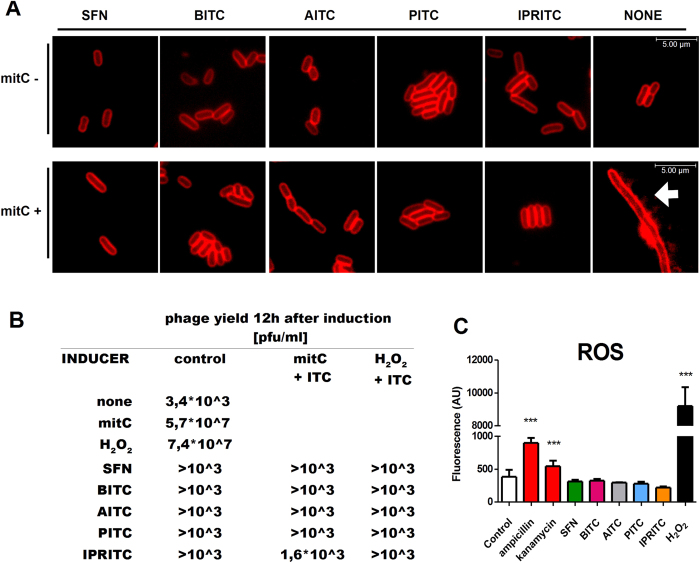
ITCs affect the prophage induction. The effects of ITCs and prophage induction by mitomycin C or hydrogen peroxide were assessed by the microscopic analysis (**A**), by phage yield (**B**) and by production of reactive oxygen species (**C**). (**A**) For microscopic analysis, prophage induction was provoked by addition of mitomycin C (1 μg/ml), ITCs (as indicated) were added at the same time. After 3 hours, samples were stained with SynaptoRed to visualize bacterial membranes. The white arrows mark filamentous *E. coli* cells, (**B**) phage yield was measured 12 hours after induction by mitomycin C (1 μg/ml) or hydrogen peroxide (1 μM). ITCs at the concentrations of 1/8, as determined for each ITC, were added at the time of induction, (**C**) reactive oxygen species level was assessed after prophage induction with hydrogen peroxide, or presence of indicated ITCs or antibiotics. The experiments were repeated independently at least 3 times. The differences among the results were examined by the T-student test. Differences with statistical significance are indicated by asterisk above columns indicating P value < 0.001.

For the potential application of ITCs as therapeutic agents against EHEC infection it is very important to assess whether the tested compounds could induce lytic development of prophages in the course of bacterial growth inhibition. Thus, we tested the effect of ITCs on the Shiga toxin-converting prophage induction. We used mitomycin C and hydrogen peroxide, two factors used to induce lambdoid prophages under laboratory conditions. Interestingly, none of the tested ITC alone induced prophage development (i.e. it was below 10^3^, the spontaneous induction level, Fig. 3B). Moreover, the effects of the chemically forced induction by mitomycin C (at the final concentration of 1 μg/ml) and H_2_O_2_ (1 μM) were severely diminished by the presence of relatively low concentration of ITCs (1/8 MIC) (Fig. 3B). Interestingly, the low level of induction, similar to the one observed for the control, was shown for all ITCs tested, regardless of their effect on bacterial growth - even for IPRITC, when the concentration of 1/8 MIC did not significantly inhibit bacterial growth, the phage induction level did not exceed 10^3^control level. Prophage induction leads to a severe disturbance of cell physiology and eventually - to cell lysis. One of the indicators of phage induction is defect in DNA replication and cell division, resulting in the filamentous morphology of bacterial cells in the presence of inducing factor, mitomycin C (Fig. 3A, panel indicated “none”). However, presence of ITCs, even at as low concentration as 1/8 of MIC, notably prevented the cellular morphology disturbance in the presence of mitomycin C (Fig. 3A).

The action of hydrogen peroxide, as well as many other chemical compounds, leads to the production of reactive oxygen species (ROS), and by damaging DNA, induces the SOS response, and subsequently, prophage development. The role of ITC in ROS formation and DNA damage still remains unclear. Thus, we tested the level of ROS during the treatment with ITC and showed that none of the tested ITCs provoked production of ROS (Fig. 3C), while significant ROS level was detected in the control experiments. As a control we used not only hydrogen peroxide but also certain antibiotics (kanamycin and ampicillin), to avoid the possible amplification of the signal by the former one (as reported in^29^).

### The ITC activity involves induction of the stringent response

**Figure 4:**
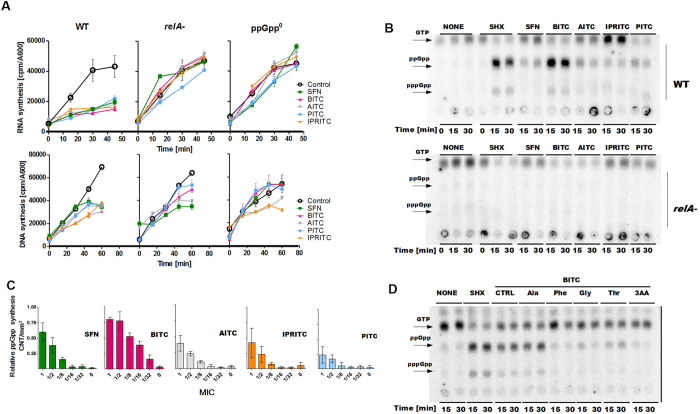
Effect of ITCs on nucleic acids metabolism. (**A**) DNA and RNA synthesis were assessed in *E. coli* wild type, Δ*relA* and Δ*relA ΔspoT* (ppGpp^0^) strains (as indicated above each panel) in the absence (empty circles) or presence of ITCs: SFN (green), BITC (magenta), AITC (grey), IPRITC (yellow), PITC (light blue) at subinhibitory concentrations (1/32,1/16,1/16,1/8,1/8 MIC respectively). The relative nucleic acid synthesis was measured by the level of incorporation of radioactive-labeled substrates, [^3^H]thymidine or [^3^H]uridine and presented as counts per minute (cpm) normalized to cell growth (A_600_). ITCs were added at time zero. The results are mean values from three independent experiments done in duplicate, with error bars indicating SD. (**B**) The synthesis of the stringent response alarmones, ppGpp and pppGpp was assessed by culturing the the wild type or *relA* bacteria in the presence of [^32^P]orthophosphoric acid (150 μCi/ml) followed by cell lysis and nucleotide separation by thin-layer chromatography. ITCs or SHX were added at time zero. Samples were withdrawn at 15 and 30 min after the addition. The positions of ppGpp and pppGpp are indicated by arrows. (**C**) The relative (p)ppGpp synthesis at 30 min after addition of indicated ITCs quantified by densitometry using phosphorimager (Typhoon, GE Healthcare) and Quantity One software. The results are the average of three independent experiments ± SD, normalized by setting the ppGpp level obtained in SHX treatment as 1. (**D**) The (p)ppGpp synthesis was assessed as in (**B**) with the addition of the amino acids (final concentration at 400 μg/ml) where indicated.

The inhibition of bacterial growth by ITCs indicates that the basic physiological and metabolic processes could be affected. Thus, we tested the ITC effect on the two major processes, transcription and replication of DNA. We found that ITCs caused an abrupt inhibition of stable RNA synthesis - the level of RNA decreased dramatically already during first minutes of ITC treatment (Fig. 4A). The effect on DNA synthesis, however, was not visible until longer treatment, and it was not very strong. All tested ITCs showed similar effects on RNA and DNA synthesis. Interestingly, the inhibition of transcription was dependent on the ability to synthesize (p)ppGpp - in the bacteria devoid of (p)ppGpp (due to mutations in *relA* and *spoT* genes) no effect of ITCs on RNA synthesis was observed. Moreover, this effect was also abolished when only *relA* gene was nonfunctional (Fig. 4A middle panel). RelA enzyme is responsive to amino acid starvation, so its dysfunction abolishes the response to SHX treatment (serine starvation). This indicates that the mode of action of isothiocyanates involve stringent response. Thus, we assessed the synthesis of ppGpp and pppGpp during ITC treatment. The level of ppGpp and pppGpp was measured after 15 and 30 minutes of addition of the indicated ITC, or serine hydroxamate (SHX), the amino acid analog used as a positive control in laboratory to induce serine starvation. We found that all tested ITCs induce synthesis of both, ppGpp and pppGpp (Fig. 4B). Interestingly, the level of accumulated ppGpp was proportional to the concentration of ITCs used in the experiment (Fig. 4C). The full chromatograms are presented in the Fig. S1. The extent of induction varied for different ITCs, when compared to the level of ppGpp observed during the induction with SHX, the lowest was observed for PITC. The ITC-mediated induction of (p)ppGpp synthesis was abolished in mutants devoid of RelA function (Fig. 4B, lower panel). This evidence indicates that ITC effect is linked to the stringent response induction via RelA-mediated pathway of (p)ppGpp synthesis.

The important question to address was: how ITCs induce the stringent response. The dependence on *relA* function indicates that the mechanism involves interfering with amino acid metabolism. Our previous studies showed that the addition of excess of certain amino acids to the bacteria treated with PEITC resulted in their growth restoration^15^. Thus, we supplemented bacterial cultures grown in the minimal medium devoid of amino acids, with 20 mM of individual amino acids. We found that in the presence of certain amino acids the sensitivity to ITCs was significantly decreased. The amino acids that could restore growth of ITC-treated bacteria and allow bacterial growth in the presence of ITCs at MIC are listed in the Table 2. The other amino acids, even at a higher excess, did not improve bacterial growth. Next, we tested whether ppGpp accumulation was affected by the addition of amino acids. We chose BITC for this test because of its strongest antimicrobial effect. In the presence of 400 μg/ml phenylalanine, glycine or threonine ppGpp synthesis was severely inhibited, while alanine did not cause any effect. Addition of all three amino acids (Phe, Gly, Thr) resulted in even stronger inhibition of ppGpp accumulation (Fig. 4D).

### ITCs inhibit *stx* gene expression and reduce toxicity of Shiga toxin-producing *E. coli* strains

**Figure 5:**
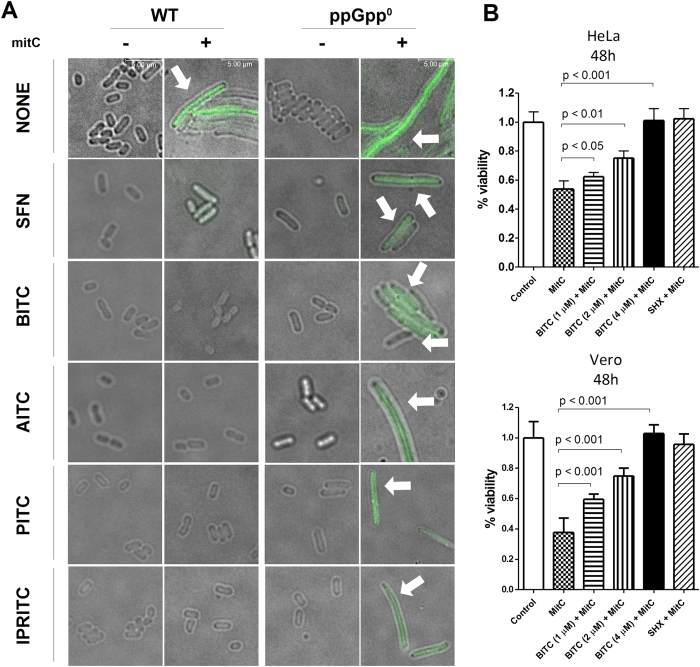
ITCs inhibit *stx* gene expression and toxicity of EHEC lysates. (**A**) The prophage induction by mitomycin C (1 μg/ml) in the presence or absence of ITCs (as indicated) was assessed for *E. coli* wild type and ppGpp^0^ strains lysogenized with 933 W phage. GFP fluorescence corresponds to the expression of *stx* genes (indicated by arrow). (**B**) Effect of BITC and SHX on viability of HeLa and Vero cells treated with EHEC strain lysate. The Shiga-toxin harboring prophage was induced in *E. coli* 86–24 strain by mitomycin C (1 μg/ml) in the absence (control) or presence of various BITC concentrations or 0.5 mg/ml SHX.

The induction of the stringent response during ITC treatment was responsible for the inhibition of not only bacterial growth, but also for inhibition of the prophage induction in Shiga toxin-harboring bacteria. As demonstrated previously, (p)ppGpp down-regulates the lambdoid phage replication and *stx* expression^14^. Thus, we tested the effect of ITCs employed in this study on this process. The lysogen of phage 933 W with the GFP-encoding gene instead of an *stx* gene was used to monitor the ITC influence on prophage induction. Mitomycin treatment caused the disturbance in cell division as a result of phage development (characteristic filamentous phenotype). The GFP synthesis was clearly visible as an indication of expression of genes in the region encoding *stx* in the wild type phage (Fig. 5A). The addition of ITCs resulted in reduction of both effects - the cell morphology returned to normal, and the GFP expression was undetectable (Fig. 5A). The effect of all tested ITCs was similar. However, the presence of ITCs during mitomycin C treatment of (p)ppGpp-devoid cells resulted in full extent of effects, corroborating the hypothesis that effective stringent response induction is indispensable for ITC effect.

The most direct effect of the induction of *stx* harboring prophages and Shiga toxin production is toxicity for mammalian cells. As we showed, ITCs can inhibit bacterial growth with no effect on prophage induction, and thus no effect on Stx production. To check whether EHEC strain treated with ITCs is toxic, we performed *in vivo* cytotoxity tests using bacterial lysates and reference strains, Vero and HeLa, and assessed their viability after exposure to bacterial lysates. Under conditions of mitomycin C-mediated prophage induction, viability of Vero and HeLa cells decreased to about 50%. A simultaneous addition of BITC reduced the toxicity of bacterial lysates; at the concentration of 4 μM the viability of mammalian cells treated with EHEC lysates reached 100% (Fig. 5B). The same effect was observed when SHX was used. Thus, we conclude that the induction of the stringent control was responsible for abolishing the toxicity of lysates of EHEC treated with BITC.

**Figure 6:**
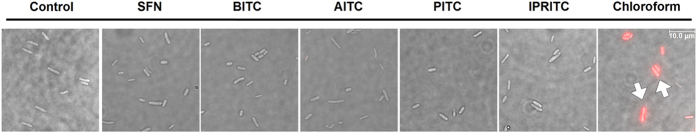
The effect of ITCs on cellular membrane integrity. Bacteria were treated with indicated ITCs at growth inhibitory concentrations, and with propidium iodide; the fluorescence signal was visualized by fluorescence microscopy. Cells with damaged membranes were marked with white arrows.

A previously suggested mechanism of antibacterial effects of selected ITCs was proposed to act through disturbance of membrane integrity^19^,^30^. The induction of the stringent response may, as an indirect effect, lead to the changes in membrane properties. Thus, we assessed the membrane integrity using propidium iodide test. Interestingly, the fluorescence signal indicating the disruption of membrane integrity was visible only for the chloroform treated control. None of the ITCs employed in our studies, at concentrations up to MIC, affected the cell membrane properties (Fig. 6). These results indicate that the ITC antimicrobial effect does not need to involve the membrane damage.

## Discussion

The number of new antibacterial compounds developed in recent years is dramatically low and discovery of new classes of such substances takes decades^31^. Thus, a problem of life threatening infections becomes increasingly serious in both, the developing and developed countries. The expectation for modern compounds with antibiotic properties is not only for their effectiveness against bacterial growth, but also for minimal side effects possible, and, what is very important, the low level of selection of strains resistant to these therapeutics. It is particularly important nowadays, when the arms race between pharmaceutical development and bacterial evolution seems to favor microorganisms. In the light of acquiring antibiotic resistance among bacteria, as well as special precautions that have to be undertaken in the case of some specific infections, the need to find new antibacterial compounds has increased. It is particularly important for EHEC infections, where conventional treatment may lead to serious medical complications and increased mortality. In our work, we exploited the possibility to employ isothiocyanates, which are plant-derived compounds, to inhibit virulence of the EHEC strains. Some of the ITCs are well known for their antioxidant, anti-inflammatory and chemopreventive properties^17^,^32^. However, very limited evidence was reported to date about antimicrobial properties of ITC, especially relating to the molecular mechanisms of action and identifying cellular targets. So far, their antibacterial effect was demonstrated for such bacterial pathogens as *E. coli*, *Staphylococcus aureus, Salmonella montevideo* and *Campylobacter jejuni*^19^,^20^,^21^,^33^. We have demonstrated previously that one of ITCs, phenetyl isothiocyanate (PEITC), efficiently inhibits growth of *E. coli* EHEC strains, and, more importantly, at the same time does not induce prophages, and thus Shiga toxin production is not induced^15^. In this report, we used a selected set of ITCs with various chemical properties and structures, to assess their potential antimicrobial effect and to search for molecular mechanisms of bactericidal activity of these plant-derived substances. We show that all tested ITCs exhibit an antimicrobial activity, and among them BITC and AITC are the most effective. The antimicrobial effect of ITCs would be useful for treatment of EHEC infections only if the bacterial growth inhibition does not result in the induction of the prophages. We found that the presence of ITCs not only inhibits bacterial growth, but also inhibits the *stx*-harboring prophages’ lytic development and production of Stx toxin. ITCs affect nucleic acid synthesis, especially RNA production, which was down-regulated shortly after ITC addition. Strong and rapid inhibition of stable RNA synthesis indicates that the global regulatory mechanism of the stringent response may be involved. The effectiveness of this mechanism relies on the short reaction time to the onset of the stress in order to preserve as much energy as possible under unfavorable conditions. When (p)ppGpp could not be synthesized due to mutations in the genes responsible for its metabolism (*relA* and *spoT*), the effect of ITCs on RNA synthesis is abolished. Thus, the dependence of RNA synthesis inhibition on the presence of functional stringent response indicates that the ITC antibacterial action indeed induces this global regulatory system. In addition, the effect of ITC on the accumulation of ppGpp was abolished in bacteria harboring only the *relA* mutation. This indicates that the mechanism of stringent response induction by ITCs involves the pathway and stress response related to amino acid starvation. Some ITCs were shown to bind to amino acids and proteins^28^,^34^. This would suggest that either the diminished availability of certain amino acids, or any other disturbance of protein synthesis, is a common mode of action for all tested ITCs. The induction of the stringent response leads then to the down-regulation of stable RNA synthesis and many other effects resulting in growth inhibition. Inhibition of DNA synthesis, although visible, was not so evident as RNA synthesis inhibition, moreover the effect on DNA synthesis was significantly delayed. Importantly, the slow-down of DNA synthesis was only partially dependent on ppGpp (Fig. 4A) suggesting that the ITC effect expands beyond the induction of the stringent response. In agreement with this, we observed that ITCs exerted their antimicrobial effect also in bacteria devoid of ppGpp (Table S1). This clearly indicates that the induction of the stringent response is an emergency strategy to increase the chances to survive during ITC treatment. The ITCs causing either simple amino acid starvation or more complex disturbances in the biomolecules synthesis affect even more severely cells defective in the stringent response, because they lack the global regulatory and defense system mediated by ppGpp. In the wild type strain, the main advantage of the induction of the stringent response by ITCs is that the growth inhibition of EHEC does not lead to the induction of *stx*-harboring prophages, due to the inhibitory effect of (p)ppGpp on bacteriophages’ main promoters and lytic development.

Our previous work on PEITC had shown that the mechanism of this ITC action involves some interaction with amino acids^15^. One possible mechanism would be binding of ITCs to amino acid residues, sequestering them from either transport to bacterial cell or from forming the aminoacylo-tRNA. It cannot be excluded, though, that ITCs affect the activity of specific tRNA aminoacylo transferases. ITCs effects lead to the induction of the stringent response in the cells capable of ppGpp accumulation via the pathway related to the amino acid starvation. However, the interaction with specific amino acids have to be considered - the list of amino acids that could reverse the antibacterial effect varied for different ITCs (Table 2). There were some amino acids common for specific ITCs, such as glycine for SFN, BITC and AITC, arginine for SFN, PITC and IPRITC, phenylalanine for BITC and AITC or lysine for PITC and IPRITC. These observed correlations do not seem to be related to the chemical structure of a given ITC, ruling out the hypothesis of specific interactions with amino acid residues. However, more detailed studies on the modeling of the binding sites of ITCs and amino acids or proteins are necessary for evaluation of the ITC interactions with cellular targets. Interestingly, the addition of amino acids that reversed the ITC effect, led to the abolishing of ppGpp accumulation (Fig. 4D). Importantly, the antimicrobial effects of ITCs were observed in rich media (LB, M-H) (Fig. 2, Table S1). This indicates that in the standard laboratory conditions of bacterial growth, even in relatively high abundance of amino acids, ITCs can inhibit bacterial growth and this effect could be reversed only in the high excess of a specific amino acid.

A previously suggested mechanism of antibacterial effect of ITCs assumed that the main targets are cellular membranes, and the disruption of their integrity underlies bacterial growth inhibition. However, in our experiments assessing membrane integrity, we found no effect of any tested ITCs, while the chloroform control was positive. It would indicate that the induction of the stringent response is mainly responsible for all growth inhibitory effects. The observed contradictory results could arise from different laboratory methods used in previous works. Method employing propidium iodide are considered as reliable and reproducible way to assess membrane integrity^35^,^36^. Certainly, it cannot be fully excluded that the previously suggested mechanisms of ITC action could result from a delayed secondary effect of the induction of the stringent response.

Bacterial virulence is usually correlated with the presence of the stringent response^37^ thus our results could seem contradictory to this observation. However, during ITC treatment in the wild type cells, the level of (p)ppGpp increases dramatically, leading not only to the down-regulation of multiple cellular processes which promotes survival, but most importantly, to inhibition of Stx production in Shiga toxin-converting *E. coli* strains. In case of the normal growth, a moderate level of (p)ppGpp, needed for growth rate regulation^38^ , is necessary for efficient expression of virulence factors or their functionality, while the significantly elevated (p)ppGpp level results in the opposite effect, the decrease in the toxicity of bacterial strains (as confirmed by toxicity test on Vero and HeLa cell lines, Fig. 5B). Even if the antibacterial effect of a given ITC is not spectacular when comparing to conventional antibiotics, the observation that induction of prophages by factors like mitomycin C or hydrogen peroxide was inhibited by the presence of ITCs (Figs 3B and 5A), suggests that ITCs could be used in addition to an antibiotic treatment to prevent Stx toxin-converting phages induction and toxin production. The possible synergistic action of ITCs and antibiotics would be a desirable aim, however one has to take into the account that the elevated level of ppGpp may lead to increased antibiotic resistance^39^,^40^,^41^,^42^ resulting from decreased amount of cellular antibiotic targets. Therefore this possibility would require extensive further investigation.

The interesting question is whether the strength of the stringent response induction and the level of (p)ppGpp synthesis does correlate with the antibacterial effect of ITCs, or whether just the induction, and even relatively low level of (p)ppGpp, is sufficient for halting of growth. IPRITC showed the weakest effect in bacterial growth test and also induced the relatively low level of (p)ppGpp synthesis. Similarly, the second of the weak inhibitors, PITC (as assessed by growth inhibition in liquid medium), does induce (p)ppGpp synthesis, but this induction is not as potent as observed for SHX or, e.g. BITC, and it is also delayed in time (Fig. 4B, upper panel). The phenyl isothiocyanate is an artificial isothiocyanate, which may underlie its lower impact on bacterial growth and stringent response induction. However, antibacterial effect of PITC was already reported^20^. The antimicrobial effect of IPRITC was not shown so far, but there were reports solely on isolation of this compound, among others, from cruciferous plants and the antibacterial effect of multicompound extracts^43^. Thus, it could be concluded that the level of (p)ppGpp is important for effective growth inhibition of bacteria. However, the effect of various ITCs on phage induction or cellular morphology upon mitomycin C treatment was similar, regardless of the differences in the growth inhibition or levels of (p)ppGpp synthesis.

Even if the antibacterial effect of a given ITC is not spectacular when comparing to conventional antibiotics, the observation that induction of prophages by factors like mitomycin C or hydrogen peroxide was inhibited by the presence of ITCs (Figs 3B and 5A), suggests that ITCs could be used in addition to an antibiotic treatment to prevent Stx toxin-converting phages induction and toxin production.

For the potential use of ITCs to treat bacterial infection, in particular EHEC infection, it has to be considered whether ITCs are safe for eukaryotic cells. Isothiocyanates are known for their chemopreventive effects, e.g. in laboratory animals, ITCs, including BITC, effectively inhibited tumorigenesis induced by carcinogens in various organs, such as colon, esophagus, lung, mammary, pancreas^44^,^45^, especially when given during or prior to carcinogen exposure. For instance, protective effects of pretreatment with BITC have been reported for benzo(a)pyrene-induced mouse lung cancer^46^. On the other hand, it has been shown that BITC might be carcinogenic if given for a long time or applied after post-initiation period. For instance, diet containing 0.1% BITC applied for 32 weeks had promoting effect on urinary bladder carcinogenesis induced by diethylnitrosamine and N-(4-hydroxybutyl)nitrosamine in rats^47^,^48^. However, human relevance of the multiorgan rat carcinogenesis models applied by these studies is unclear. Nevertheless, toxicological evaluations of long-term administration of BITC in different species, including human, are needed to assess the comparative risk and benefit of its use.

To summarize, we present evidence that isothiocyanates, which are plant-derived compounds, exhibit antibacterial activity that in the wild type cells leads to the induction the stringent response. Notably, the growth inhibition of EHEC strains does not lead to the induction of *stx*-harboring prophages and to production of Shiga toxins due to ppGpp accumulation. Thus, ITCs could be considered as potential therapeutic agents in EHEC infections.

## Additional Information

**How to cite this article**: Nowicki, D. *et al.* Isothiocyanates as effective agents against enterohemorrhagic *Escherichia coli*: insight to the mode of action. *Sci. Rep.* **6**, 22263; doi: 10.1038/srep22263 (2016).

## Supplementary Material

Supplementary Information

## Figures and Tables

**Table 1:** *Escherichia coli* strains used in this work.

*E. coli* strain	Relevant genotype	Reference
*E. coli* K-12 laboratory strains		
MG1655	F^-^ λ^-^ *ilvG rfb-*50 *rph*	49
CF1652	As MG1655 but *relA251*::*kan*	50
ppGpp^0^	As MG1655 but *relA256 spoT212*	14
SCUC34	A 933 W Δ*stx2*::*catGFP* lysogen	51
MG1655 (933 W)	MG1655 lysogenic with 933 W Δ*stx2*::*catGFP*	14
*E. coli* O157:H7 clinical isolates		
86–24	O157:H7 lysogenized with ST2-8624	52
CB571	O157:H7 stx1, stx2	53

**Table 2:** Amino acids decreasing the sensitivity of bacteria to ITCs as determined by MIC.

Isothiocyanate	Amino acids
SFN	Gly, Cys, Arg, Trp, Asp
BITC	Gly, Phe, Thr
AITC	Gly, Phe, Thr, Trp, Asp, Gln
PITC	Arg, Lys
IPRITC	Arg, Lys, Glu
