# Supplementary material for: Isothiocyanates as effective agents against enterohemorrhagic Escherichia coli: insight to the mode of action
Source: Sci Rep. 2016 Feb 29;6:22263. doi: 10.1038/srep22263 (PMC4770323; doi:10.1038/srep22263)

Isothiocyanates as effective agents against enterohemorrhagic *Escherichia coli*: insight to the mode of action

Dariusz Nowicki, Olga Rodzik, Anna Herman-Antosiewicz, Agnieszka Szalewska-Pałasz*

Table 1S. Effect of ITCs on growth of wild type and stringent response deficient bacterial strains. MIC was determined for each ITC according to Andrews (2001) microdilution assay. The bacteria were cultured in minimal (MOPS MM) or rich medium (M-H). M-H: Mueller-Hinton medium (acid hydrolysate of casein, 17.5 g/l, beef extract, 2 g/l, starch, 1.5 g/l). The results are from at least three independent experiments done in duplicates.

MIC mg/ml(mM)									
ITC\ strain	MG1655			*relA*$^-$			ppGpp^0		
medium	M-H	MOPS MM + 0,2% Casa	MOPS MM	M-H	MOPS MM + 0,2% Casa	MOPS MM	M-H	MOPS MM + 0,2% Casa	MOPS MM
SFN	0.71 (4)	0.18 (1)	0.09 (0.5)	0.35 (2)	0.18 (1)	0.18 (1)	0.18 (1)	0.18 (1)	0.09 (0.5)
BITC	0.07 (0.5)	0.07 (0.5)	0.02 (0.125)	0.07 (0.5)	0.04 (0.25)	0.02 (0.125)	0.04 (0.25)	0.04 (0.25)	0.02 (0.125)
AITC	0.40 (4)	0.40 (4)	0.20 (2)	0.40 (4)	0.40 (4)	0.20 (2)	0.20 (2)	0.20 (2)	0.1 (1)
PITC	0.27 (2)	0.52 (4)	0.14 (1)	0.27 (2)	0.52 (4)	0.27 (2)	0.27 (2)	0.27 (2)	0.27 (2)
IPRITC	0.81 (8)	0.81 (8)	0.41 (2)	0.81 (8)	0.81(8)	0.41 (4)	0.41 (4)	0.41 (4)	0.41 (4)

Figure 1S. The induction of the stringent response by increasing amounts of ITCs. The synthesis of the stringent response alarmones, ppGpp and pppGpp was assessed by culturing bacteria in the presence of [^{32}P]orthophosphoric acid (150 μCi/ml) followed by cell lysis and nucleotide separation by thin-layer chromatography. ITCs (as indicated above each panel) or SHX were added at time zero (lanes 1,3) and samples were withdrawn at 15 (lanes 2,4,7,9, 11,13,15) and 30 minutes (lanes 3,5,8,10,12,14,16) after the addition. ITCs were added the final concentrations of 1 x MIC (lanes 7,8), 1/2 MIC (lanes 9,10), 1/8 MIC (lanes 11, 12), 1/16 MIC (lanes 13,14), 1/32 MIC (lanes 15,16). The positions of ppGpp, pppGpp, GTP, GDP and ATP are indicated by arrows.

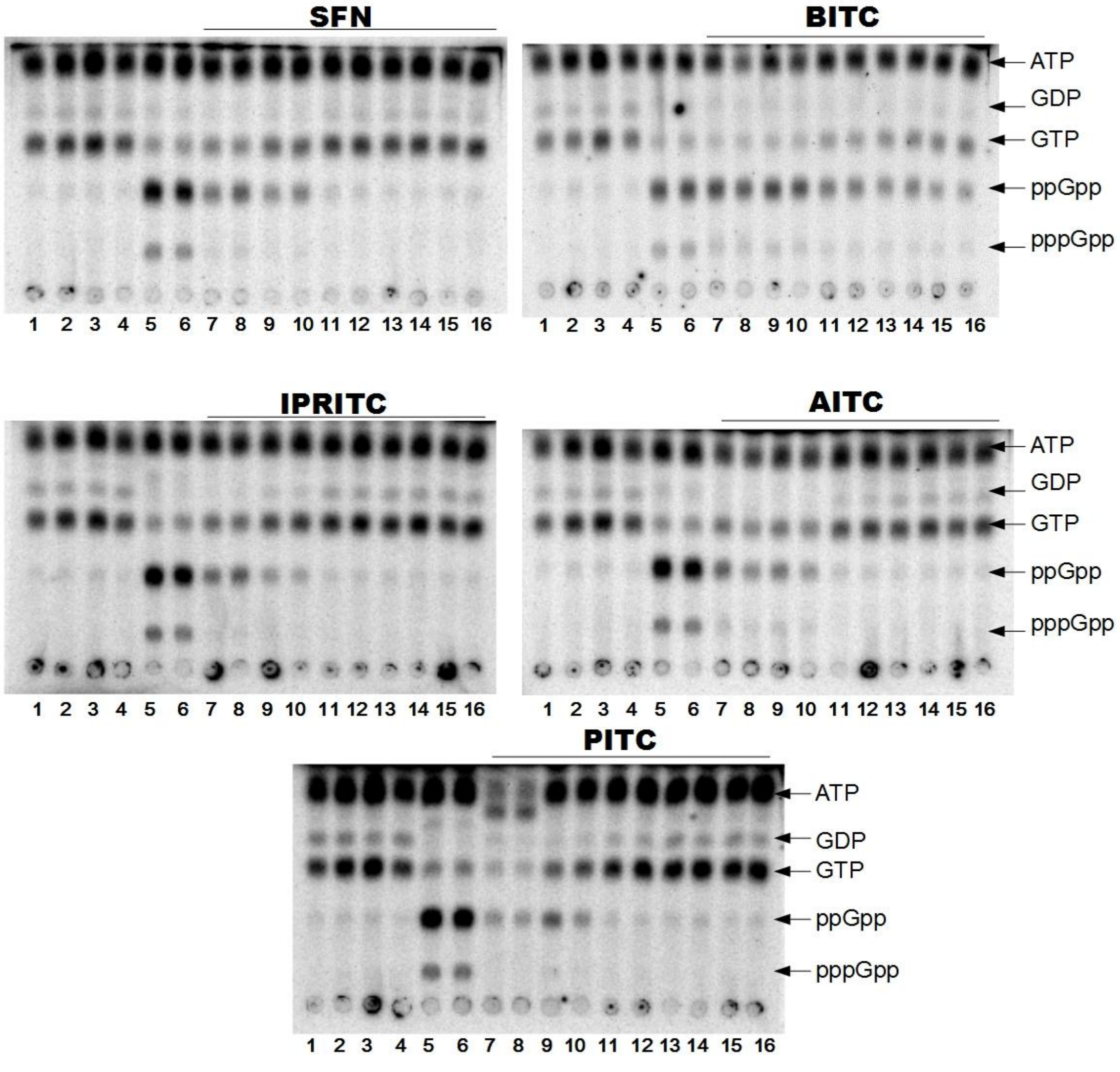

Supplement: Supplementary Information [file srep22263-s1.pdf]
